# Cold acclimation alleviates cold stress-induced PSII inhibition and oxidative damage in tobacco leaves

**DOI:** 10.1080/15592324.2021.2013638

**Published:** 2021-12-29

**Authors:** Yanli Wei, Hongzhi Chen, Lu Wang, Qin Zhao, Di Wang, Tongen Zhang

**Affiliations:** Institute of Biological Engineering, Xinxiang Institute of Engineering, Xinxiang, Henan, China

**Keywords:** Tobacco, cold acclimation, cold stress, osmotic regulation, antioxidant mechanism, photoprotective

## Abstract

This study aimed to explore how cold acclimation (CA) modulates cold stress in tobacco leaves and reveal the relationship between CA and cold stress resistance, and the mechanism of CA-induced plant resistance to cold stress. This study examined the effects of CA treatment (at 8–10℃ for 2 d) on the cold tolerance of tobacco leaves under 4°C cold stress treatment using seedlings without CA treatment as the control (NA). In both CA and NA leaves, cold stress treatment resulted in a decrease in maximum photochemical efficiency of PSII (*F*_v_*/F*_m_), increase in relative variable fluorescence (*V*_J_) at 2 ms on the standardized OJIP curve, inhibition of PSII activity, and impairment of electron transfer on the acceptor side. Besides increasing the malondialdehyde (MDA) content and electrolyte leakage rate, the cold stress exacerbated the degree of membrane peroxidation. The CA treatment also induced the accumulation of reactive oxygen species (ROS), including superoxide anion (O_2_·^−^) and H_2_O_2,_ and increased the activities of antioxidant enzymes, such as superoxide dismutase (SOD), peroxidase (POD), catalase (CAT) and ascorbic acid peroxidase (APX). The CA treatment also enhanced the accumulation of soluble sugar (SS) and soluble protein (SP), cyclic electron flow (CEF), and the proportion of regulatory energy dissipation Y(NPQ). Moreover, CA+ cold stress treatment significantly reduced CEF and Y(NPQ) in tobacco leaves than under NA+ cold stress treatment, thus significantly alleviating the degree of PSII photoinhibition. In conclusion, CA treatment significantly alleviated PSII photoinhibition and oxidative damage in tobacco leaves under cold stress treatment. Improvement in cold resistance of tobacco leaves is associated with the induction of antioxidant enzyme activity, accumulation of osmoregulation substances, and initiation of photoprotective mechanisms.

## 1. Introduction

Cold stress is one of the most common meteorological disasters in agricultural production in alpine areas. Exposure of plants and animals to lower suboptimal temperatures prior to cold stress condition modulates their physiological processes inducing cold stress resistance, a process known as cold acclimation (CA).^1^The CA treatment modulates morphological, anatomical, physiological and biochemical properties of plants, significantly enhancing their stress resistance.^2^ Plants acclimate to cold stress by accumulating osmotic regulators, such as soluble sugar (SS),^3^ proline (Pro),^4^ soluble protein (SP), and polyamines (PAs).^5^ Plants exhibit enhanced activities of antioxidant enzymes, such as superoxide dismutase (SOD), peroxidase (POD), catalase (CAT), glutathione peroxidase (GPX), and ascorbic acid peroxidase (APX) and eliminate excess reactive oxygen species (ROS) to alleviate the oxidative damage in plant cells under cold stress^6–9^ Plants can also resist cold injury by increasing the proportion of unsaturated fatty acids (oleic acid, linoleic acid, and linolenic acid),^10,11^ CA is induced by highly complex biochemical mechanisms, including the expression of genes encoding stress proteins (dehydrins), increased levels of sugars, enhanced antioxidant defense mechanisms, and changes in lipid compositions^12–14^ CA increases the proportion of unsaturated fatty acids in cucumber seedlings, improves fluidity, and alleviates the damage of membrane peroxidation under cold stress by improving the activity of antioxidant enzymes and accumulating antioxidants in plants.^15^ CA also induces plant cold resistance by regulating the metabolism of hormones, such as gibberellin, cytokinin, and auxin^16^ or inducing the expression of potential anti-freeze genes or proteins^17–19^ Cold stress can regulate about 20% of the genes in the genome of the model plant *Arabidopsis thaliana*.^20^ Cold stress also induces Ca^2+^ channels, increases intracellular Ca^2+^ concentration, regulates the phosphorylation or dephosphorylation of proteins, thus initiating the expression of genes related to cold stress and synthesis of cold-resistant specific proteins, thus enhancing cold resistance of plants.^21^ Cunningham et al.^22^ found that 2℃ cold stress treatment can induce the expression of inositol galactoside synthase gene (*GaS*) in alfalfa and the *GaS* gene expression in root cap tissues of alfalfa varieties with strong cold resistance. Stockinger et al.^23^ showed that *CBF1* gene induced the expression of the cold regulated/responsive (CORs) genes and enhanced the cold resistance of *Arabidopsis thaliana*. Similarly, other researchers have also indicated that *CBF* family genes can induce the overexpression of CORs genes and improve the cold resistance of transgenic plants.^24,25^ Moreover, the production of endogenous anti-freeze proteins (AFP) in *Secale cereale* L. through CA can improved the cold resistance of ryegrass (*Secale cereale* L.).^26^

Photosynthesis is highly sensitive to cold stress.^27^ Cold stress decreases the photochemical activities^28–30^ inhibits carbon assimilation,^31,32^ causes the imbalance between plant light energy absorption and utilization,^33^ and perturbs the ROS metabolism, especially for thermophilic plants.^34,35^ Plants have developed various photosynthetic regulatory mechanisms to adapt to cold stress during the long-term evolution process. For instance, the damage of D1 protein in plant PSII reaction center under cold stress can be reassembled through rapid turnover.^36^ Plants can dissipate excess light energy through non-chemical quenching (NPQ) and other ways to prevent excessive reduction of Q_A,_ thus balancing the absorption and utilization of light energy.^37,38^ Particularly, the energy quenching component (*qE*) in NPQ can dissipate the absorbed light energy into heat energy.^39^ CA can prevent photodamage under cold stress and promote recovery.^40,41^ For instance, CA can improve the PSII photochemical activity of mulberry under cold stress.^42^ CA can also increase the activity of many Calvin cycle enzymes, including Rubisco.^43^ Moreover, CA significantly enhances the synthetic ability of D1 in cucumber seedlings than in the NA plants.^44^ CA can also improve the photosynthetic ability of plants under cold stress by regulating hormones and redox signals in chloroplasts.^45^

For tobacco production in high latitude areas, seedlings are raised in a greenhouse, and then transplanted to the field in early spring, when outside temperature may drop to 2–4°C. The transplanted seedlings undergo cold stress in the fields, resulting in growth retardation and poor survival rate. For this reason, part of the greenhouse plastic film is often uncovered before the seedlings are transplanted to ensure the temperature is maintained at about 8–10℃ for CA treatment, thus improving the cold stress resistance of tobacco seedlings. The seedlings are then transplanted into the field after several days of CA treatment, as a measure to prevent the effect of cold stress (2–4℃) after transplantation, and significantly enhancing seedling survival and growth. The seedlings without cold acclimation (NA) may be at risk of chilling and freezing stress. This study assessed the changes of osmotic regulatory substance accumulation and antioxidant enzyme activity in tobacco seedlings under CA treatment (8–10℃) and 4℃ cold stress to reveal the internal mechanism of this phenomenon. The mechanism of cyclic electron flow (CEF) and energy distribution of PSII reaction center in improving the activity of PSII reaction center under cold stress was also studied. This study can provide a theoretical basis for improving the cold stress tolerance of tobacco seedlings and formulating appropriate cultivation measures.

## 2 Materials and methods

### 2.1 Plant materials and treatment

Tobacco seeds were sown in peat soil and vermiculite matrix at a fully mixed volume ratio of 1:1. One seedling was retained in each bowl (length, width, and height, 7.5 cm). The seedlings were raised in a greenhouse at 25℃ and a light intensity of 400 μmol·m^−2^·s^−1^, photoperiod of 12/12 h (light/dark). Forty seedlings with relatively consistent growth were selected and divided into two groups of 20 seedlings each. Seedlings in one group were put in an artificial climate box at 8–10℃ for cold acclimation treatment (recorded as CA). The seedlings in the other group were grown in the greenhouse without cold acclimation (NA). The CA and NA seedlings were placed in a 4℃ low-temperature artificial climate box for cold stress treatment after 2 d of CA treatment. Except for temperature, other cultural and environmental conditions were the same as normal growth conditions during CA and cold stress treatments. The physiological indexes were measured after CA and cold stress treatments for 2 d.

### 2.2 Determination parameters and methods

#### 2.2.1. Determination of OJIP curve

The tobacco leaves with different treatments were subjected to 0.5 h dark adaptation using a dark adaptation clamp. A Handy-PEA chlorophyll fluorometer (Hansatech company, UK) was used to assess the OJIP curve of leaves after the dark adaptation. Each treatment had five replicates, and the OJIP curve was drawn using the average value of the five repetitions. The O, J, I, and P points on the OJIP curve were 0.01, 2, 30, and 1000 ms, respectively. The relative fluorescence intensities of O and P points were expressed in *F*_o_ and *F*_m,_ respectively. The maximum photochemical efficiency of PSII (*F*_v_/*F*_m_) was then calculated as follows: *F*_v_/*F*_m_ = (*F*_m_*-F*_o_)/*F*_m_. The OJIP curve was standardized using the formula, *V*_O-*P*_ = (*F*_t_-*F*_o_)/(*F*_m_-*F*_o_), to obtain the *V*_O-P_ curve, and the relative variable fluorescence *V*_J_ of point J at 2 ms on the *V*_O-P_ curve, where *F*_t_ represents the relative fluorescence intensity of each time point on the OJIP curve. The difference between the standardized *V*_O-P_ curve and NA treatment curve of tobacco seedling leaves under different treatments was expressed as Δ*V*_O-P_.

#### 2.2.2. Determination of PSII energy distribution parameters

The tobacco leaves with different treatments were subjected to 0.5 h dark adaptation using a dark adaptation clamp. The FMS-2 chlorophyll fluorometer (Hansatech company, UK) was used to measure the initial fluorescence (*F*_o_) and maximum fluorescence (*F*_m_) after dark adaptation. The steady-state fluorescence (*F*_s_) was also measured under the light adaptation of 1000 μmol·m^−2^·s^−1^. The PSII effective quantum yield Y(II), PSII non-regulated energy dissipation Y(NO), and PSII regulated energy dissipation yield Y(NPQ) were calculated using the following formulae: Y(II) = (*F*_m_-*F*_s_)/*F*_m_, Y(NO) = *F*_s_/*F*_m_, and Y(NPQ) = 1-Y(II)-Y(NO).^46^

#### 2.2.3. Proton Gradient Regulation (PGR5)-dependent cyclic electron transfer (PGR5-CEF) measurement

The treated leaves were adapted to the dark for 30 min, treated with 20% weak far-red light treatment for 60 s to oxidize P700. The far-red light was switched off to reduce P700 in the dark, then the same intensity of weak far-red light was given for 60 s. Finally, 100% saturated far-red light (1000 μmol·m^−2^·s^−1^) was irradiated for 1 s, while the saturated actinic light (5000 μmol·m^−2^·s^−1^) was on. The maximum signal of P700 was then measured. The M-PEA (Hansatech, UK) was then used to measure the redox kinetics of the pigment molecule of the PSI reaction center (P700). Only the proton gradient promotes ATP formation during far-red light treatment, thus significantly reducing the P700 signal. The P700 signal increases when the far-red light is turned off due to the presence of cyclic electron transfer, and the rising gap indicates the level of PGR5-CEF.^47^ Each treatment had three biological replicates.

#### 2.2.4. NDH-dependent cyclic electron transfer (NDH-CEF) measurement

FMS-2 portable modulated fluorometer (Hansatech, UK) was used to measure the change of chlorophyll fluorescence signal. The leaves were adapted to dark for 30 min, then irradiated with 54 μmol·m^−2^·s^−1^ light. The light was turned off at 300 ms. The chlorophyll fluorescence signal increased due to the NDH-dependent CEF. The difference between the lowest and highest points was used to qualitatively describe NDH-CEF.^48^

#### 2.2.5. Determination of physiological indexes (ROS metabolism and osmoregulation substance content)

The generation rate of O_2_·^−^ and H_2_O_2_ content were determined as described by Yang et al. (2021).^49^ A conductivity meter (DDS-11 C) was used to determine the electrolyte leakage rate, expressed as relative conductivity. The content of malondialdehyde (MDA), soluble sugar (SS), and soluble protein (SP) was determined as described by Wang.^50^ Superoxide dismutase (SOD), peroxidase (POD), catalase (CAT) and ascorbate peroxidase (APX) activities were measured using the appropriate kits (Suzhou Comin Biotechnology Co., Ltd. (Jiangsu, China)). The activity (1 U) of SOD was defined as the amount of enzymes required to reduce nitrotetrazolium blue chloride (NBT) to half of that of the control group. The activity (1 U) of CAT was defined as a 0.1 absorbance reduction at 240 nm. POD activity (1 U) was defined as a 0.01 absorbance reduction at 470 nm. The activity of APX was defined as the amount of ascorbic acid (AsA) (μmol) oxidized per g of fresh sample per min. The activity of dehydroascorbate reductase (DHAR) was defined as the amount of AsA (μmol) per g of fresh sample per min.

### 2.3 Data processing and statistical methods

Excel and SPSS (12.0) software were used for all data analysis. The data of each index in the figure were expressed as the mean ± standard deviation (SE) of the three replicated samples. ANOVA followed by the LSD test was used to compare the differences among the different treatments.

## 3 Results and analysis

### 3.1 Plant phenotype

**Figure 1.**
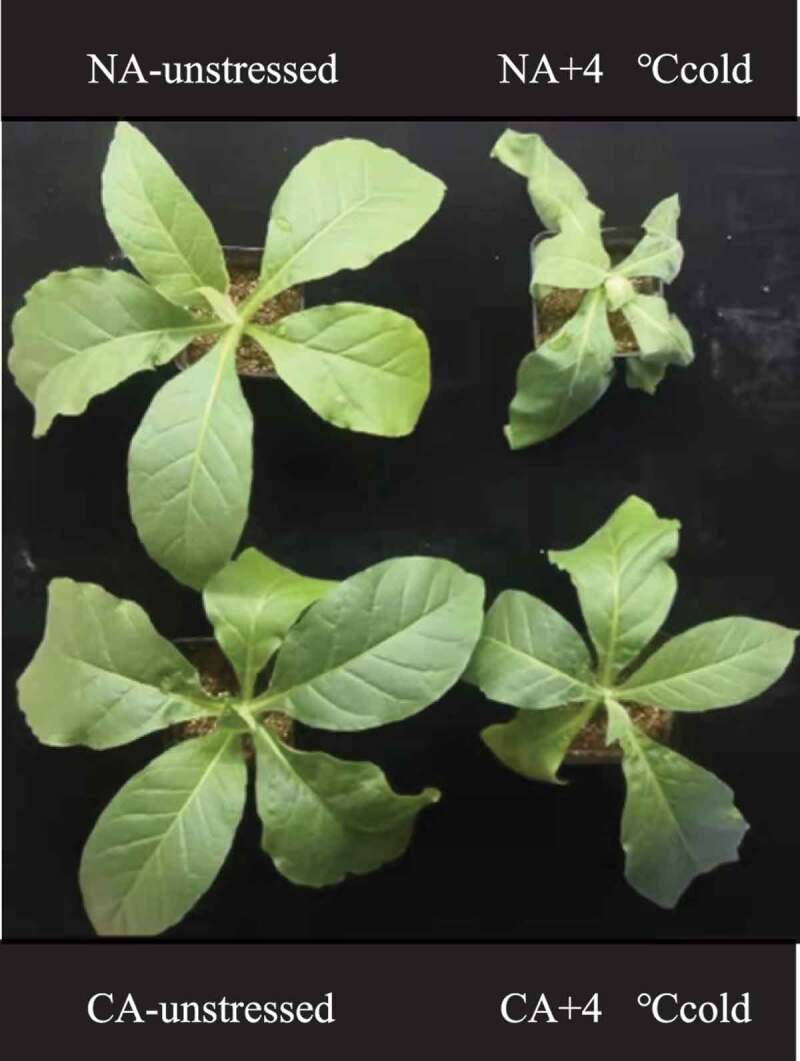
Effects of cold acclimation on plant phenotype of tobacco seedlings under 4℃ cold stress.

The CA treatment caused slight wilt in tobacco leaves, but it did not show significant symptoms of chilling injury compared with the NA group (Figure 1). The NA+ cold stress treatment significantly wilted and shrunk the tobacco leaves. However, the CA+ cold stress treatment had significantly less severe symptoms of chilling injury in tobacco, and the leaves maintained an upright position.

### 3.2 OJIP curve and PSII photochemical activity

**Figure 2.**
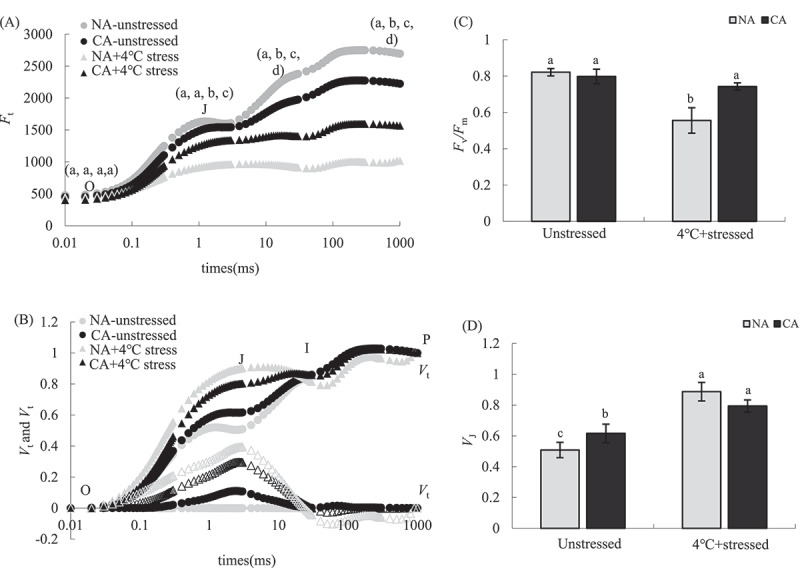
Effects of cold acclimation on OJIP curve (a), standardized OJIP curve (b), *F*_v_*/F*_m_(c) and *V*_J_ (d) of tobacco seedling leaves under 4℃ cold stress.

Note. The data in the figure is the average value of three replicated samples. Different letters indicate significant differences among different treatments at *P* < .05.

In general, cold stress reduced the relative fluorescence of OJIP curves in both CA and NA plants. The differences between the CA and NA group under stressed and unstressed treatments widened significantly over time. The OJIP curve of tobacco seedling leaves treated with CA had no significant change in the relative fluorescence intensity at points O and J compared with NA (*P* > .05) (Figure 2a). In contrast, the relative fluorescence intensity at points I and P was significantly lower in the CA group than in the NA group (*P* < .05). CA increased the relative variable fluorescence *V*_J_ of point J on the *V*_O-P_ curve based on the standardization of OJIP curve (*V*_O-P_) of tobacco seedling leaves under different treatments (Figure 2b). The cold stress treatment significantly decreased the relative fluorescence intensity at each point on the OJIP curve, while it increased *V*_J_ (Figure 2d). However, the changes in OJIP curve and *V*_J_ were less in the CA+cold stress group than in the NA+cold stress group. Their *F*_v_*/F*_m_ was not significantly different between the CA and NA groups without stress treatment, but the CA leaves had significantly higher *F*_v_/*F*_m_ than the NA (*P* < .05) after the cold stress treatment (Figure 2c). However, *V*_J_ increased by 21.22% (*P* < .05) in the CA group compared with the NA group. The cold stress treatment significantly decreased *F*_v_*/F*_m_ while it increased *V*_J_ in NA plants. However, *F*_v_*/F*_m_ was 33.71% higher in the CA+ cold stress group than in the NA+4°C group (*P* < .05), while *V*_J_ was 10.50% lower in the CA+ cold stress group than in the NA+4°C group (*P* < .05) (Figure 2c,d).

### 3.3 Energy distribution parameters of PSII reaction center

**Figure 3.**
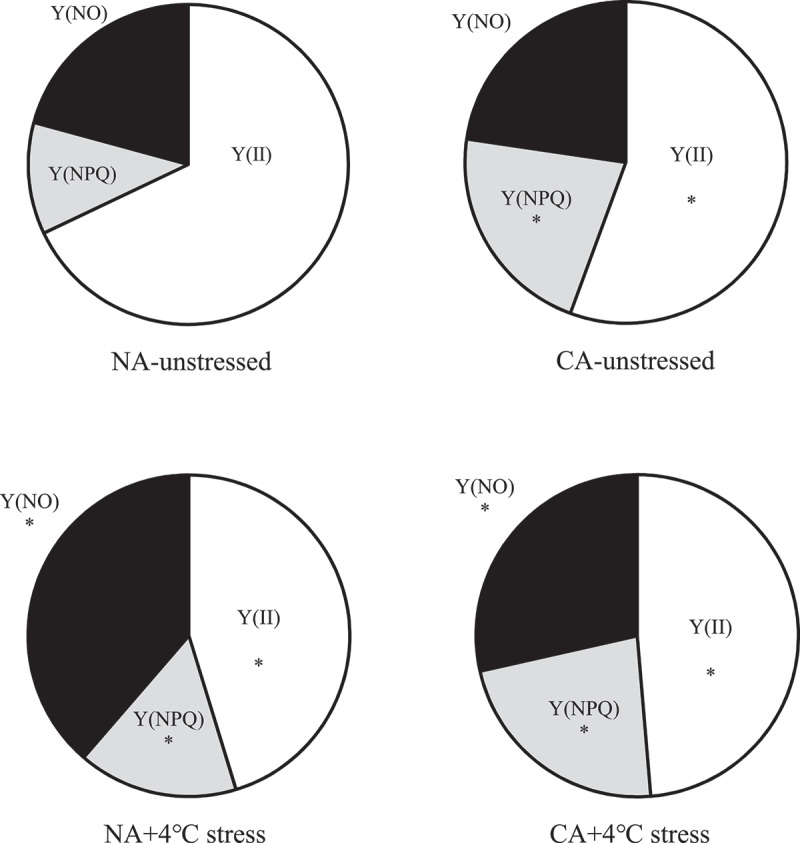
Cold acclimation on Y(II), Y(NPQ) and Y(NO) with and without cold stress in tobacco leaves.

Note. The data in the figure is an average of three replicated samples. *Showed significant difference between treatment and NA-unstressed (*P* < .05), ns showed no significant difference between treatment and NA-unstressed (*P* > .05).

The leaf Y(II) of the CA group showed a significant decreasing trend compared with that of NA under un-stressed condition (Figure 3), mainly due to the increased Y(NPQ) (increased by 10.41% compared with the NA group). The Y(NO) only increased by 1.91% in the CA group compared with the NA group without cold stress treatment. The cold stress treatment significantly decreased Y(II) (*P* < .05), while it increased Y(NO) (*P* < .05). However, Y(II) was significantly higher in the CA+ cold stress group than in the NA+ cold stress group, while Y(NO) was significantly lower in the CA+ cold stress group than in the NA+4°C. Moreover, the Y(NPQ) was slightly higher in the CA+ cold stress group than in the NA+ cold stress group by 6.74%.

### 3.4 Cyclic electron flow

**Figure 4.**
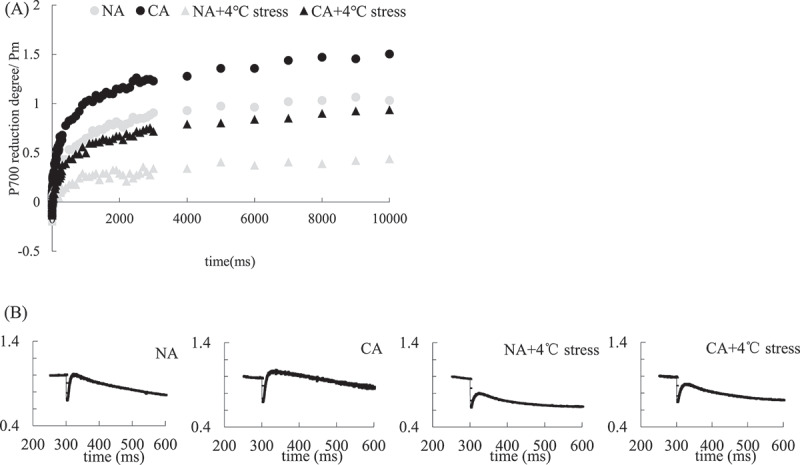
Effect of cold acclimation on PGR5-passway (a) and NDH-passway (b) CEF under cold stress in tobacco leaves.

Note. The data in the figure is the average of three replicated samples.

CA treatment significantly increased the rapid rise of chlorophyll fluorescence (NDH-pathway CEF) and the rise drop of the signal after turning off far-red light (PGR5-pathway CEF) compared with the NA group (Figure 4). The cold stress treatment decreased the CEF of both NDH-pathway and PGR5-pathway in all treatment groups. However, the magnitude of decrease of CEF in both pathways was less in the CA+ cold stress group than in the NA+ cold stress group.

### 3.5 ROS content and membrane peroxidation

**Figure 5.**
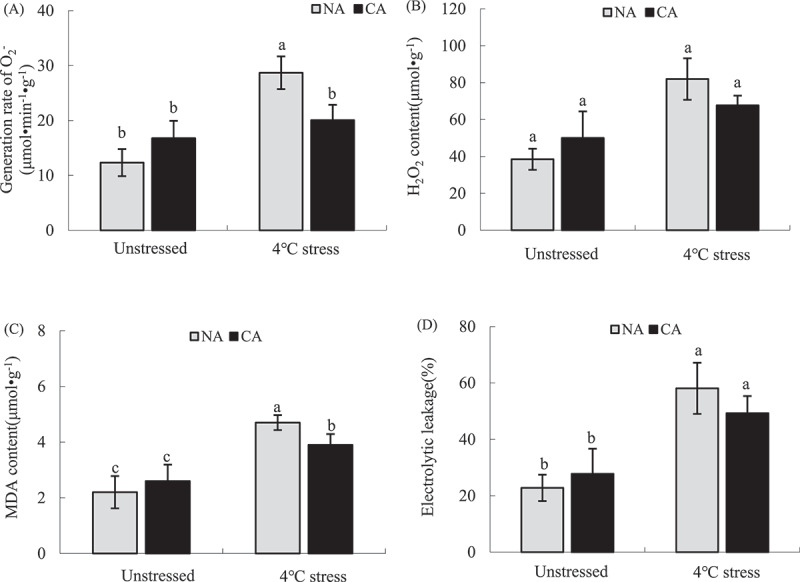
Effect of cold acclimation on generation rate of O_2_^−^ (a), H_2_O_2_ content (b), MDA content (c) and electrolytic leakage (d) with and without cold stress in tobacco leaves.

Note. The data in the figure is the average value of three replicated samples. Different letters indicate significant differences among different treatments at *P* < .05.

CA slightly increased the generation rate of O_2_·^−^ and H_2_O_2_ content in the tobacco leaves (Figure 5). However, the generation rate of O_2_·^−^ and H_2_O_2_ content were not significantly different between the NA and CA groups prior to cold stress treatment. Moreover, CA slightly increased the MDA content and electrolyte leakage rate compared with the NA group prior to exposure to cold stress. The cold stress significantly increased ROS content and the degree of membrane peroxidation in both CA and NA plants. However, the generation rate of O_2_·^−^, H_2_O_2_ and MDA contents and electrolyte leakage rate were lower in the CA+ cold stress group than in the NA+ cold stress group by 30.09% (*P* < .05), 17.43% (*P* > .05), 17.02% (*P* < .05), and 12.92% (*P* > .05), respectively.

### 3.6 Antioxidant enzyme activity

**Figure 6.**
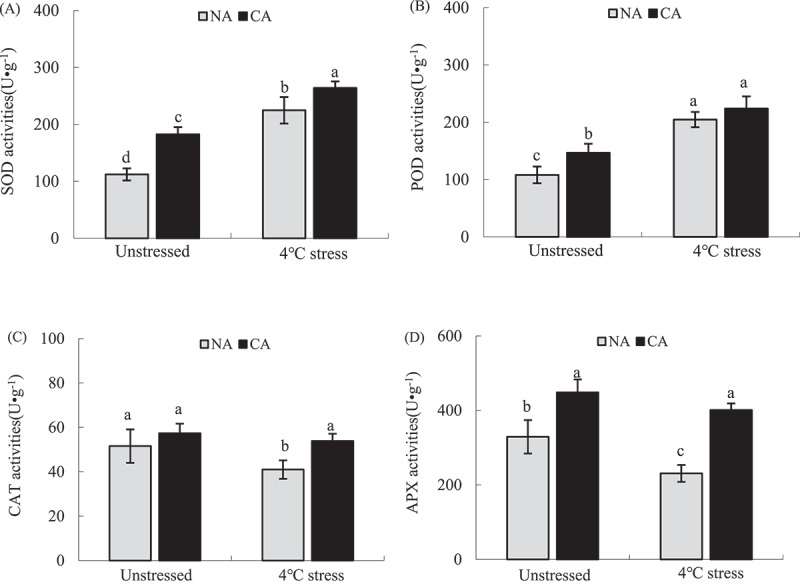
Effect of cold acclimation on SOD (a) POD (b), CAT (c) and APX (d) activity under cold stress in tobacco leaves.

Note. The data in the figure is the average value of three replicated samples. Different letters indicate significant differences among different treatments at *P* < .05.

CA significantly increased the activities of SOD, POD, and APX in tobacco leaves, compared with the NA group without cold stress treatment (Figure 6). The cold stress treatment significantly increased SOD and POD activities, while it decreased APX activities and CAT showed relatively stable in both CA and NA plants. However, SOD, CAT, and APX activities were significantly higher in the CA+ cold stress group than in the NA+ cold stress.

### 3.7 Osmoregulation substances

**Figure 7.**
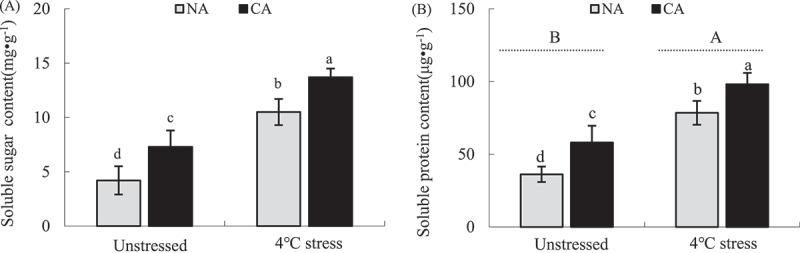
Effect of cold acclimation on soluble sugar (a) and soluble protein (b) content under cold stress in tobacco leaves.

Note. The data in the figure is the average value of three replicated samples. Different letters indicate significant differences among different treatments at *P* < .05.

CA increased SS and SP contents in tobacco leaves by 73.81% (*P* < .05) and 60.49% (*P* < .05), respectively, higher than those in NA treatment (Figure 7). Moreover, the cold stress significantly increased SS and SP contents in both NA and CA plants. However, the contents of SS and SP were higher in the CA+ cold stress group than in the NA+ cold stress group by 30.48% (*P* < .05) and 25.09% (*P* < .05), respectively.

Figure 8

**Figure 8.**
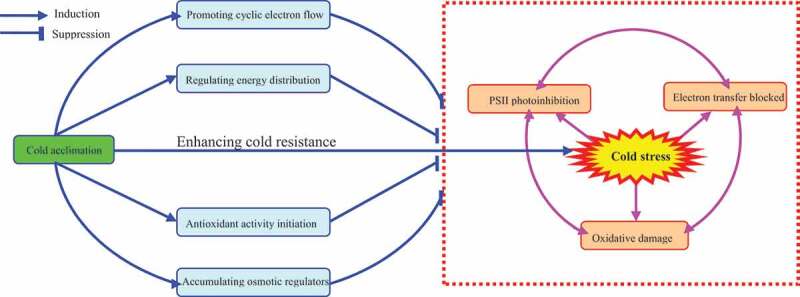
Mechanisms of cold acclimation on enhancing cold tolerance of tobacco leaves.

### 3.8 Correlation between photosynthetic and physiological parameters

As can be seen from, *F*_v_/*F*_m_ was negatively correlated with *V*_J_, O_2_^−^, H_2_O_2_, MDA and EL (*P* < .05), and positively correlated with CAT activity (*P* < .05). The *V*_J_ parameter was positively correlated with O_2_^−^, H_2_O_2_, MDA, EL, SOD, POD, SS, and SP (*P* < .05), and negatively correlated with CAT and APX (*P* < .05).Table 1

**Table 1.** Correlation between photosynthetic and physiological parameters of tobacco leaves under different treatments

	*F*_v_/*F*_m_	*V*_J_	O_2_^−^	H_2_O_2_	MDA	EL	SOD	POD	CAT	APX	SS	SP
*F*_v_/*F*_m_	1.00											
*V*_J_	−0.88*	1.00										
O_2_^−^	−0.97*	0.95*	1.00									
H_2_O_2_	−0.92*	1.00*	0.97*	1.00								
MDA	−0.92*	0.99*	0.96*	0.99*	1.00							
EL	−0.89*	0.99*	0.93*	0.99*	1.00	1.00						
SOD	−0.54	0.87*	0.70	0.83	0.81*	0.84*	1.00					
POD	−0.66	0.93*	0.78	0.90*	0.90*	0.92*	0.98*	1.00				
CAT	0.89*	−0.62*	−0.76	−0.67	−0.71	−0.67	−0.16	−0.33	1.00			
APX	0.77	−0.45*	−0.60	−0.51	−0.56	−0.52	0.03	−0.16	0.98*	1.00		
SS	−0.51	0.85*	0.65	0.80	0.80	0.84*	0.99*	0.98*	−0.17	0.12	1.00	
SP	−0.52	0.86*	0.67	0.81*	0.81*	0.84*	0.99*	0.99*	−0.17	0.25	0.99*	1.00

* indicates a significant correlation (*P* < 0.05) between the two parameters.

## 4. Discussion

Cold stress inhibits PSII photochemical activity in plant leaves.^51,52^ The degradation of D1 protein blocks the electron transfer on the PSII receptor side under cold stress, causing excessive reduction.^53^ Studies have shown that the degree of PSII photoinhibition linearly decreases with decreasing temperature (25–4°C).^54^ The relative variable fluorescence *V*_J_ at point J at 2 ms on the standardized OJIP curve represents Q_A_^−^ accumulation of the reduced primary electron receptor.^55^ Increased *V*_J_ indicates that the blocking of electron transfer flow from Q_A_^−^ to Q_B_ leads to the excessive reduction of Q_A_^−^, and thus is an important index of blocked electron transfer on the PSII receptor side.^56,57^ In our study, CA treatment increased *V*_J_ in tobacco leaves, but it did not significantly decrease *F*_v_/*F*_m_. The *F*_v_/*F*_m_ indicates the activity of the PSII reaction center.^58^ These results show that CA treatment did not cause PSII photoinhibition in tobacco seedling leaves. However, the cold stress treatment decreased *F*_v_/*F*_m_. At the same time, it significantly increased *V*_J_, thus significantly blocking electron transfer on the PSII receptor side and leading to substantial photoinhibition of the PSII reaction center. However, the degree of PSII photoinhibition was significantly lower in the CA+ cold stress group than in the NA+ cold stress group. These results indicate that CA can significantly alleviate PSII photoinhibition in tobacco leaves under 4°C cold stress and promote the electron transfer from the PSII reaction center.

Plants produce excess energy than their utilization capacity under stress.^59,60^ This results in a decrease in the photosynthetic rate and electron transport capacity, thereby undergoing photoinhibition.^61^ Enhanced non-chemical quenching (NPQ) is essential in dissipating the excess energy in PSII, thus alleviating PSII photoinhibition.^62–64^ The PSII-regulated energy dissipation yield Y(NPQ) reflects the ability of PSII to convert excess excitation energy into heat through regulatory energy dissipation (xanthophyll cycle-related energy dissipation).^65^ Herein, CA treatment significantly decreased Y(II) and increased Y(NPQ) and Y(NO) compared with the NA group, especially for Y(NPQ). These results show that although CA treatment can reduce PSII photochemical activity in tobacco leaves, the seedlings can adapt to CA through regulatory energy dissipation, preventing excessive reduction of plastoquinone pool leading to photoinhibition of the PSII. However, severe stress can gradually aggravate PSII photoinhibition if the excess excitation energy is not consumed through the NPQ pathway and other electron sinks in time, thus producing more ROS that destroy the photosynthetic mechanism.^66,67^ Moreover, the adaptive mechanism of regulatory energy dissipation is often destroyed under severe oxidative damage.^68^ The Y(NO) represents the non-regulatory energy dissipation of PSII, which refers to the ability of PSII to passively dissipate excitation energy by closing the reaction center of PSII due to photoinhibitory damage. It can also reflect the photooxidative damage of PSII.^69,70^ Herein, cold stress reduced the ability of tobacco leaves to dissipate excess energy through the NPQ pathway compared with the CA treatment. However, Y(NPQ) was significantly higher in the CA+ cold stress group than in the NA+4°C group. Moreover, Y(II) was significantly higher in the CA+ cold stress group than in the NA+4°C group. In contrast, Y(NO) was significantly lower in the CA+ cold stress group than in the NA+ cold stress group. These results show that CA treatment can improve resistance to cold stress by regulating the energy distribution of the PSII reaction center.

The CEF is an important photoprotection mechanism in plants when under stress.^71,72^ Increased CEF rate under stress can inhibit the attack of excess electrons on O_2,_ thus reducing oxidative damage.^73^ Although CEF cannot produce NADPH, it can be produced by a proton pump. The ΔpH drives ATP synthesis and increases ATP/NADPH ratio.^74,75^ The additional ATP can also resist various adversity^76^ and prevents PSI and PSII photoinhibition.^77,78^ Moreover, the CEF activity in plants caused by cold stress is known.^79,80^ CEF pathway mainly involves NAD(P)H dehydrogenase (NDH) and Proton Gradient Regulation 5/Proton Gradient Regulation-Like 1 (PGR5/PGRL1) pathways.^81,82^ Studies have shown that plant CEF is mainly mediated by the PGR5 pathway,^76^ while the protective effect of the NDH pathway is weak under cold stress.^83^ However, this study showed that CA treatment increased PGR5 and NDH pathways to varying degrees. CEF can also favor the repair of core protein D1 of PSII under stress, promote the linear electron transfer process,^84^ increase NPQ and alleviate the photoinhibition of PSII.^85–87^ Therefore, CA treatment can promote PSII receptor side electron transfer (Figure 2) and NPQ dependent regulatory energy dissipation mechanism (Figure 3) in tobacco leaves under cold stress, possibly because CA can promote CEF in tobacco leaves.

Soluble sugar (SS) and soluble protein (SP), key osmoregulation substances during adverse conditions, can resist dehydration and reduce the damage of cold stress to cells.^88,89^ Studies have shown that CA can improve the cold tolerance of mulberry leaves by promoting the accumulation of organic substances, such as SS and proline,^90^ similar to this study. Herein, CA significantly increased SS and SP contents in tobacco leaves than in the NA group. However, SS and SP contents were significantly higher in the CA+ cold stress group than in the NA+ cold stress group (Figure 7). CA promoted the accumulation of SS and SP in tobacco leaves, thus improving cold stress tolerance.

Cold stress and other stresses significantly inhibit the physiological processes, such as photosynthesis and respiration of plants. Excess energy and electrons can mediate the ROS production, thus intensifying the cell membrane lipid peroxidation^91–93^ As a result, plants often alleviate the peroxidation damage caused by ROS by increasing antioxidant enzymes or accumulating antioxidants.^94,95^Excess O_2_·^−^ in cells can be reduced through SOD,^96,97^ CAT, and POD scavenge excess H_2_O_2_in cells.^98–100^ However, H_2_O_2_ in chloroplasts mainly depends on APX clearance in the AsA-GSH cycle.^101,102^ CA slightly increased O_2_·^−^ and H_2_O_2_ contents in tobacco leaves compared with the NA group. Moreover, CA had a less inhibitory effect on PSII activity in tobacco leaves (Figure 2). CA also increased SOD, POD, CAT, and APX activities (Figure 6). In addition, the correlation analysis showed that the change of CAT activity was the main positive factor affecting *F*_v_/*F*_m_. Cold stress significantly increased SOD and POD activities in tobacco leaves, while it inhibited APX and CAT activities, increasing ROS content and the accumulation of membrane lipid peroxidation product MDA. Studies have shown that although enzymes in plant cells can remove excess ROS, they are vulnerable to ROS attack and lose their activity under severe stress,^103,104^ Therefore, the cell oxidative damage and PSII photoinhibition in tobacco leaves under the cold stress could be due to the inhibition of antioxidant enzyme activities, such as CAT and APX. Herein, SOD, POD, CAT, and APX activities were higher in the CA+ cold stress group than in the NA+ cold stress group, indicating that CA enhances antioxidant enzyme activity and prevents inactivation under severe stresss, thus improving cold tolerance.

The mechanism of how CA improves the cold tolerance of tobacco leaves is summarized in Figure 7Briefly, CA can alleviate the PSII photoinhibition and oxidative damage of tobacco leaves under 4℃ cold stress by promoting the accumulation of osmotic regulators, improving the activity of antioxidant enzymes, promoting cyclic electron transfer, and optimizing the energy distribution of PSII reaction center.

## 5. Conclusion

The cold stress leads to the inhibition of PSII activity and the obstruction of electron transfer on the receptor side. Besides increasing the MDA content and electrolyte leakage rate, the cold stress exacerbated the degree of membrane peroxidation. CA treatment increased CEF and Y(NPQ) in tobacco leaves, reducing excess excitation energy and alleviating PSII photoinhibition under 4°C cold stress. In conclusion, CA treatment (8–10°C for 2 d) can significantly reduce the degree of PSII photoinhibition and oxidative damage in tobacco leaves under 4°C cold stress. Our study can contribute to further elucidation of cold acclimation and cold stress response mechanism in tobacco and other mesophilic plants, and provide a theoretical basis for the growth of tobacco in cold areas.
